# Notes and News

**Published:** 1902-02-15

**Authors:** 


### Notes and News.

HOSPITAL MEETINGS.

Charing Cross Hospital.—Annual Meeting at the Hospital, 2 p.m.

A tramp, who has been discovered to be suffering from small-pox, has been in five or six workhouses since the time when he is believed to have contracted the disease.

Christian Scientists have opened a vigorous campaign in Berlin. It is reported that very marked disapproval of this form of quackery is being shown at the Court.

A meeting of the local branch of the National Association for the Prevention of Consumption was lately held at Winchester with a view to formulating scheme to provide a Consumption Sanatorium for the district. Mr. Malcolm Morris addressed the meeting.

A new infirmary has been opened at the Solihull Workhouse. The accommodation is for eighty patients, distributed over four large wards and several small ones for special and isolation cases. There is a pavilion for the use of convalescents and a maternity department.

The establishment of invalid kitchens after the Berlin method might well be followed in all large towns. It is a form of providence which has very much to recommend it, and little to fear from abuse. It should be worked with the co-operation of parish and other doctors, and could usefully be managed by a staff of ladies who have leisure for such work.

It is a common failing of humanity to underrate blessings, and few things cause more anger than enforced salutary measures. Not very long ago Mr. Long was one of the best detested of men, especially amongst the ladies of England. This was when every dog in the kingdom wandered muzzled and harmless amongst us. The result to-day is that rabies is practically non-existent, and its terrors are nearly forgotten. A glance at statistics from the Buda Pesth Pasteur Institute shows a very different state of affairs, where no Mr. Long formulates unpopular but beneficial regulations. No less than 2,093 persons bitten by mad animals were treated in one year. All that the Hungarian Government proposes to do is to build another institute.

A bed has been endowed to the memory of Queen Victoria at the Cornelia Hospital, Poole.

Two new operating theatres are being constructed at the Liverpool Royal Infirmary, the gift of an anonymous donor.

The small-pox outbreak near Swansea has shown itself in the town itself, and shows signs of spreading in South Wales generally. There is an immense demand for vaccination, and extra stations are to be opened.

What should prove a very useful society has recently been formed in Paris. This is a League against Infant Mortality. The methods of the league will be largely educational, but a systematic visitation of homes and institutions is to be made, and the milk supply carefully watched.

It is reported that in the operation recently performed to separate the Hindoo twins, in Paris, the cinematograph was used to record the operation. Whether this was for popular or scientific purposes, we do not know, but judging from the unnatural speed which is generally observable in cinematograph representations, the result as illustrating an operation must often be very deceptive.

There are undoubtedly difficulties in the way of general practitioners when called upon to diagnose small-pox, seeing that the symptoms are often varied or obscure, and that doctors have rarely opportunities of studying the disease at all. To facilitate matters, Dr. Dudfield, Medical Officer of Health for Kensington, proposed to the Metropolitan Asylums Board that they should place the services of an expert at the call of general practitioners for the purpose of assisting them in the diagnosis of doubtful cases. The Board pointed out that such a course might cause undue delay in notification, and so declined to adopt the suggestion. But to assist in the spread of necessary knowledge they will offer, with the permission of the Local Government Board, greater facilities for the study of small-pox at their institutions than have hitherto been available.

In July last Dr. William Osler, Professor of Medicine, Johns Hopkins University, Baltimore, offered to present the Norfolk and Norwich Hospital a silver casket, which he considered would be a suitable and proper receptacle in which to deposit the skull of Sir Thomas Browne, the eminent Norwich physician, that it might be reverently preserved and protected. The Museum Committee of the institution were pleased to accept so generous an offer, but suggested that the casket should be formed, not of silver, but of plate glass, thus allowing the skull to be well seen on all sides and at all times. Early in December the casket was presented by Mr. C. Williams in the name of Professor Osler to the Museum Committee, who were gratefully pleased to accept the very handsome gift, and they directed that an appropriate pedestal should be made on which the gift was ordered to be permanently placed. The casket is of oblong shape, about 13 inches in length by 11 inches in width and 11 inches in height. The four sides and top consist of crystal glass with silver-gilt mountings, and set on a stand of ebony.

